# Factors Associated with Time Intervals for Diagnosis of Colorectal Cancer: A Hospital Based Study in Khon Kaen, Thailand

**DOI:** 10.31557/APJCP.2020.21.6.1835

**Published:** 2020-06

**Authors:** Attapong Rittitit, Supannee Promthet, Krittika Suwanrungruang, Kriangsak jenwitheesuk, Chalongpon Santong, Patravoot Vatanasapt

**Affiliations:** 1 *Faculty of Public Health, Khon Kaen University, Khon Kaen, Thailand. *; 2 *ASEAN Cancer Epidemiology and Prevention Research Group (ACEP), Faculty of Public Health, Khon Kaen University, Khon Kaen, Thailand. *; 3 *Cancer Unit, Srinagarind Hospital, Faculty of Medicine, Khon Kaen University, Khon Kaen, Thailand. *; 4 *Department of Surgery, Faculty of Medicine, Khon Kaen University, Khon Kaen, Thailand. *; 5 *Department of Otorhinolaryngology, Faculty of Medicine, Khon Kaen University, Khon Kaen, Thailand. *

**Keywords:** Time intervals, delay diagnosis, colorectal cancer

## Abstract

**Background:**

Colorectal cancer (CRC) is among the five-leading cancers in Thailand. Delayed diagnosis is crucial for undermining the prognosis of the patients. This study aims to evaluate the factors associated with the time interval for diagnosis (TID).

**Methods:**

A cross-sectional analytical study of 191 CRC patients with histological confirmation who were undergoing treatment in the tertiary hospital in Khon Kaen Province was conducted. The data were obtained by interview and retrieving from medical records. The time interval in each diagnostic process is reported in geometric mean. The geometric mean ratio (GMR) used to interpret the results from multiple linear regressions that analyze the relationship between factors and log-transformed TID.

**Results:**

Most patients were males (61.78%) with mean age of 61.28±10.2 years old. The geometric mean of TID was 263.48 days. Two factors were significantly associated with longer TID: first visit at a tertiary hospital (GMR=7.77 relative to secondary hospital; 95%CI=1.95 to 30.57) and distance to tertiary healthcare. Two factors were significantly associated with shorter TID: officer/ state enterprise (GMR=0.53 relative to agriculture; 95%CI=0.28 to 0.98) and cost of traveling to secondary healthcare.

**Conclusions:**

The results showed the occupation, first health care visit, distance and cost were factors associated with TID. Improving the facilities at the secondary healthcare units for diagnosing CRC would be likely to help to reduce the wasted time in the healthcare system.

## Introduction

Colorectal cancer (CRC) killed over 600,000 people per year worldwide and still represents one of the most common cancer (Wu et al., 2016). The global burden of CRC is expected to increase by 60% to more than 2.2 million new cases and 1.1 million deaths by 2030 (Arnold et al., 2017). Nearly 45% of CRC cases worldwide occurred in Asia with an increasing trend of its incidence (Chiu et al., 2015).

In Thailand, colorectal cancer is the third common cancer in males (The age-standardized incidence rate (ASR) = 15.2 per 100,000 population) and the fifth in females (ASR = 10.1 per 100,000 population) (Ferlay et al., 2015). Likewise, the incidence of CRC in Thailand showed increasing trends in both sexes (Pongnikorn et al., 2015).

Waiting time is a key indicator for quality of cancer management. The report from Manitoba, overall waiting times for treatment of CRC continuously increased during the years 2001-2005 owing to diagnostic delays (Singh et al., 2010). Similarly, the study of the five most common cancers in the Netherlands showed a considerable increase about 10-25% of patient’s duration of diagnostic intervals (defined as the time from the visit of a general practitioner for cancer related symptoms to definite diagnosis). Moreover, the increasing proportion of time for primary care in CRC would increase diagnostic intervals (Helsper et al., 2017).

Delayed diagnosis related to poor outcomes of CRC, as the previous study found that who had delayed diagnosis were significant with late stage of CRC and breast cancer (Martin et al., 2007; Ermiah et al., 2012; Ortiz-Ortiz et al., 2016) , and with higher mortality of CRC patients (Tørring et al., 2011; Tørring et al., 2012). In addition, among participants who had delayed colonoscopy were associated with lower cancer-specific survival (Li et al., 2019), and patient’s delay was related to negative result of survival of non-small cell lung cancer patients (Radzikowska et al., 2012). Also, The National Survey of NHS Patients suggested that minimizing diagnostic delays to increase the proportion of early-stage cancers may improve cancer survival in the UK (Allgar and Neal, 2005). Significant studies provided that the fast-track diagnosis and treatment may be a good way for suspected cancers and access to general diagnostic (Olesen et al., 2009). 

The research implicates the factors associated with the time interval of CRC diagnosis is crucial. In Thailand, only a few studies on waiting time in cancer, particularly in CRC were done (Thongsuksai et al., 2000; Poum et al., 2014). Hence, this study aims to determine the factors associated with the time interval for the diagnosis of colorectal cancer patients in a super tertiary hospital in Thailand.

## Materials and Methods

This descriptive cross-sectional study was conducted from October 2018 to December 2018 at a department of surgery within a super tertiary hospital of Khon Kaen, Thailand. Patients who had been diagnosed with CRC including histological confirmation and were undergoing treatment at the hospital were eligible for inclusion in the study. Eligible participants who had multiple primary sites or physiological or psychological problems which affected their ability to communicate were excluded. 

**Figure 1:**
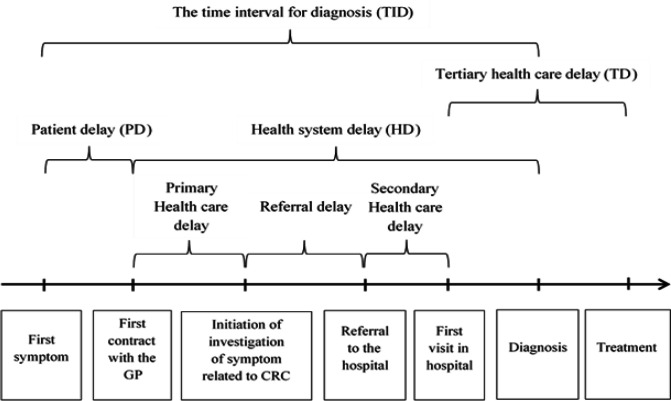
The Time Intervals in the Patients Care Process

The primary outcomes were time intervals in the diagnostic process (TID), the duration from the onset of the patient’s first symptom until date of diagnosis by histopathology (Figure 1). Other outcomes were patient delay (PD), the duration from the onset of patient’s first symptom until the first visit to the health care unit, health system delay (HD); the duration from the patient’s first visit to the health care unit to confirming the diagnosis of CRC, tertiary health care delay (TD); the duration from the patient’s first visit in the tertiary hospital until start to the first treatment. The dates involved in medical services at the hospital and the information of cancer were retrieved from the medical records.

All participants were interviewed using a structured questionnaire including the demographics, the first symptom of CRC and the date of onset. Factors associated with the time interval of diagnosis such as cost (baht), distance (km.) and time (hours.). Cost (baht), the travel expenses for public transport and private transportation calculated by fuel cost in the personal cars or expenses for rental car that travel from patient’s residence to hospital or health care center in each healthcare level. Distance (km.), the distance for traveling from patient’s residence to hospital or health care center in each healthcare level. Time (hours.), duration for traveling from patient’s residence to hospital or health care center in each healthcare level. 

The sample size required minimum of 193 patients was calculated from the formula for multiple linear regressions (Cohen, 1988). 

$f^{2}=(\frac{R_{t/c}^{2}}{1-R_{c}^{2}-R_{t/c}^{2}})$

Cohen’s determine magnitude of effect size which encloses: 0.02 = small, enclose 0.15 = medium, enclose 0.35 = large. In this study, the researcher assigned the certain effect size (f 2) nearly 0.15 is a medium. This resulted in a sample size of 193 patients.

$n=\frac{\lambda (1-R_{\mathrm{Y.A}}^{2})}{R_{\mathrm{Y.A}}^{2}}=\frac{\lambda }{f^{2}}$

*Statistical analysis*

Descriptive statistics was used to present as number with a percentage for categorical data and mean with standard deviation (SD) for continuous data. If the data was in non-normal distribution, geometric mean and median were used. 

The distribution of TID was in non-normal distribution (right-skewed) we then transformed it in natural-log-transformed. In addition, distance, cost and time of traveling were presented by log-transformed. 

Univariable linear regression was used to analyze the association between of each factor and TID. Then we fitted all the factors with a significant level on the univariable analysis (p<0.25) into the multiple regression model to identify those factors with the strongest independent effects on the outcomes. Multivariable linear regressions analysis was utilized to fit model and assess the relationship between TID and interest factors adjusting for potential confounding factors. The backward elimination method was used to construct the best model. 

The results from linear regression were presented in term of geometric mean ratio (GMR) when the outcome variable was log-transformed. These values corresponded to log-scale for difference in means gives a confidence interval for the ratio of the geometric means of the original outcome variables.

The continuous data were interpreted as the percentage increase in the dependent variable for every 1% increase in the independent variable. For all statistical tests, a p-value less than 0.05 and their 95% confidence interval was significant. Statistical analysis was performed with STATA software version 15.0.

*Ethical considerations*

This study was approved by Khon Kaen University Ethics Committee for Human Research based on the Declaration of Helsinki and the ICH Good Clinical Practice Guidelines. The reference number is HE 611258.

## Results

*Sample Characteristics*

A total number of 193 participants were recruited, with meeting the eligibility requirements and agreeing to participate. Of the 193 eligible participants, 191 were included in the study with two subjects excluded due to incomplete data. The majority were males 61.78% and married 83.77%. The mean age was 61.28 ± 10.2 years. The common first symptom presentation was constipation 111 subjects (58.12%) followed by rectal bleeding 90 subjects (47.12%) and abdominal pain 68 subjects (35.60%) (Table 1). Almost all participants initially visited the hospital instead of the primary care unit. 

*Interval Durations*

The time intervals in each cancer management process were reported in median (days) and geometric mean (days) with their 95% confidence interval. The longest interval within TID was HD, followed by PD and TD, respectively (Table 2).

*Factors Associated with TID*

With multiple linear regression, we found that the two factors associated with shorter TID were the officer/state enterprise occupation (GMR=0.53; 95%CI=0.28 to 0.98) and greater cost of traveling to secondary health care (GMR= -0.28; 95%CI= -0.55 to -0.01). On the other hand, the two factors associated with longer TID were the first health care visit to the tertiary hospital (GMR=7.77; 95%CI=1.95 to 30.57) and longer distance from home to the tertiary health care (GMR=0.33; 95%CI=0.06 to 0.60). We did not find a relationship between the number of visits at the secondary and log-transformed TID (Table 3).

## Discussion

We found that agriculture was associated with longer TID in relation to the officers or state enterprise. While agriculture is a proxy for lower education (This study showed the highest of education level was 82.26% for primary school and below). The national statistical office reported the education of Thai farmers who graduated at elementary school and secondary school or upper were 64.1% and 21.5% in 2013, respectively (The national statistical office, 2013). Therefore, the prior studies found that lower education level and lower socioeconomic status associated with delayed presentation and lower awareness of their cancer symptoms (Macleod et al., 2009; Al-Azri et al., 2017). Some previous studies about factors related to delay in the diagnostic process of CRC were inconsistent partly due to different health care contexts and different definitions of delay time (Sikdar et al., 2017; Zarcos-Pedrinaci et al., 2018). On the other hand, many evidence showed association between socioeconomic factors and time interval such as the retried female, a large household Higher, lower income, distressing and seriousness in symptom related to shorter time interval (Hansen et al., 2008; Siminoff et al., 2011; Esteva et al., 2013; Forbes et al., 2014; Oberoi et al., 2015; Walter et al., 2016; Miles et al., 2017; Windner et al., 2018).

We also found that the first health care visit at a tertiary hospital is associated with increased TID, longer waiting, when compared with a secondary hospital. One explanation for this phenomenon is most walk-in patients without referral required multiple visits for investigation instead of preceding directly for treatment as a referral case. Furthermore, additional visits may be required for referral documentation. On the other hand, several results from previous studies reported the patients who first visited at lower the hospital level were significant delayed diagnosis (Shieh et al., 2014; Frie et al., 2019).

The factors about travelling, we found that greater distance (from the patients’ house travelling to the tertiary hospital) was significantly increasing TID. One possible explanation of travelling cause of delay was the patients had inconvenienced to travel to see a specialist for screening or colonoscopy. Consequently, the cancer patients who lived a longer distance seem to have a poor result with advance of stage. As represented by longer travelling ≥50 miles were related to metastasis disease when compared with shorter distance (<12.5 miles) (Massarweh et al., 2014). The evidence about delayed diagnosis in Breast cancer, Thailand had similarly reported as our results; factors associated with significantly increasing doctor delay were travel time to the hospital (Poum et al., 2014). The consistency study to our finding, a place of residence in urban/rural associated with diagnostic delay in Breast cancer, (OR=1.72; 95% CI=1.42 to 1.93) (Dianatinasab et al., 2018). Inconsistent findings reported the CRC patients who living in a rural area, and travelling farther to a GP in urban areas, may decrease the likelihood of emergency admissions and a poor survival, it was occurring because how awareness of their patients symptoms (Murage et al., 2017). 

Surprisingly, the greater cost of travelling to secondary healthcare, which was associated with decreasing TID. Because travelling private transportation ,i.e. personal car or rental car is more expensive but faster. The result of our study found that the median of distance and cost of traveling from patient’s residence to secondary hospital and tertiary hospital were 12 kilometers (100 baht) and 90 kilometers (500 baht), respectively. These findings were consistent with the study of mode of transportation. Among participants used personal vehicles or hiring private vehicles to find the care because the public transportation was not available frequently, and they need to spend the total of 500 baht on an average for traveling (Sharma and Vong-Ek , 2012; Suprasert, 2015). 

Although, this is a hospital-based study which collected the data at the end of the virtual pathway by interview. The limitation of this study was a less precise record of the date of the first symptom presentation by interview because the patients could not remember or difficult to tell the accurate date. The problem was resolved by asking the reference date, for instance; a major Thai event/holiday or season that can help the participants to remember the event. 

In conclusion, healthcare service performance was related to diagnosis interval. If the time interval for diagnosis was to be reduced, we must focus on improving facilities of secondary hospitals to be able to diagnose of CRC to reduce the redundant visit to the tertiary hospital; and raising awareness of health care practitioners of the symptom presentation of CRC. The future research in a larger sample size in a population level would help in explaining the bigger burden perspective.

**Table 1:** Demographics and Characteristics of Colorectal Cancer Patients

Variable	Number (n=191)	Percentage
Gender		
Male	118	61.78
Female	73	38.22
Age (years)		
<40	7	3.66
40-59	16	8.38
50-59	47	24.61
>=60	121	63.35
Mean (SD)	61.28 (± 10.18)	
Median (Min:Max)	62 (24 : 89)	
Frist health care visit		
Primary health care unit	5	2.62
Secondary hospital	73	38.22
Tertiary hospital	49	25.65
Private Hospital	64	33.51
Insurance		
Nothing/Self-pay	11	5.76
The Civil Servant Medical Benefit Scheme	116	60.73
(CSBMS)		
The Social Security System (SSS)	3	1.57
The Universal Coverage Scheme (UCS)	55	28.8
Other	6	3.14
Stage of CRC		
Stage I	20	10.47
Stage II	57	29.84
Stage III	68	35.6
Stage IV	44	23.04
Unknown	2	1.05

**Table 2:** The Time Intervals along the Diagnostic Process in CRC

Variable	Number*	Median (days)	Geometric Mean (days)	95% Confidence Interval^ a^
Time intervals for diagnosis (TID)	191	268.5	263.48	227.64 to 304.98
Patient delay (PD)	189	61	74.04	59.30 to 92.43
Health system delay (HD)	183	91	98.3	79.92 to 120.90
Tertiary health care delay (TD)**	136	35	32.79	27.32 to 39.36

^a^, 95%CI of geometric mean; *The geometric mean is used for positive numbers. If the time interval has a zero or negative value. It is not being calculated; **TD, First visit tertiary hospital to first treatment received

**Table 3:** Association between Factors and the Log-Transformed Time Intervals for Diagnosis of CRC and Their 95% Confidence Intervals and Adjusted for All Covariate Factors in the Table Using Multiple Linear Regressions

Variable	N (%)	Geometric Mean (days)	Coefficients (95%CI)	GMR* (95%CI)
Occupation (n=191)				
Agriculture	60 (31.41)	272.57	Ref.	Ref.
Merchant	22 (11.52)	249.5	0.61 (-0.68 to 1.89)	1.84 (0.51 to 6.62)
Officer/ state enterprise	73 (38.22)	250.78	-0.64 (-1.27 to -0.02)	0.53 (0.28 to 0.98)
Unemployed	36 (18.85)	284.6	-0.40 (-1.03 to 0.22)	0.67 (0.36 to 1.25)
Frist health care visit (n=191)				
Secondary hospital	78 (40.84)	288.73	Ref.	Ref.
Tertiary hospital	49 (25.65)	277.46	2.05 (0.67 to 3.42)	7.77 (1.95 to 30.57)
Private hospital	64 (33.51)	226.54	-0.47 (-1.01 to 0.06)	0.63 (0.36 to 1.06)
Smoking (n=191)				
Yes	88 (46.07)	298.95	Ref.	Ref.
No	103 (53.93)	236.54	-0.41 (-0.89 to 0.06)	1.51 (0.41 to 1.06)
Distance (Km.) (n=191)				
(To tertiary health care)		71.14	0.33 (0.06 to 0.60)	0.33 (0.06 to 0.60)
Mean (SD)	117.85 (122.19)			
Median (Min:Max)	90 (1:1280)			
Cost (baht) (n=96)				
(To secondary health care)		122.90	-0.28 (-0.55 to -0.01)	-0.28 (-0.55 to -0.01)
Mean (SD)	225.52 (339.49)			
Median (Min:Max)	100 (10:2500)			
Number of visits in secondary hospital (n=114)				
1	28 (24.56)	197.90	Ref	Ref
>1	86 (75.44)	307.26	-0.01 (-0.60 to 0.57)	0.99 (0.55 to 1.77)
Number of visits in tertiary hospital (n=166)				
1	35 (21.08)	214.50	Ref	Ref
>1	131 (78.92)	285.35	-0.10 (-0.73 to 0.54)	0.90 (0.48 to 1.72)
Number of health provider visit (General doctor) (n=117)				
1	36 (30.77)	186.79	Ref	Ref
2-3	41 (35.04)	281.05	-0.38 (-1.05 to 0.30)	0.68 (0.35 to 1.35)
>4	40 (34.19)	333.59	0.18 (-0.49 to 0.85)	1.20 (0.61 to 2.34)

* Geometric mean ratio (GMR) was ratio of expected geometric mean (calculated by the exponentiated coefficient). For continuous data were interpreted the GMR as the percent increase in the dependent variable for every 1% increase in the independent variable.
